# On the Etiology of Carcinoma

**Published:** 1904-01-30

**Authors:** 


### ON THE ETIOLOGY OF CARCINOMA.

MR. KEITH W. MONSARRAT[1] reports the result of four years of research on the morphology of an organism associated with carcinoma mammæ. He claims to have found in carcinomatous tumours of the female breast an organism which will grow on nutrient media, has a definite life history, and can produce a new growth in the tissues of a living animal. Cultures can be obtained by rubbing a piece of fresh carcinomatous tissue on the surface of a glucose agar slope, or immersing it in 4 per cent. glucose broth. The growth is slight but definite, though subculture is very difficult.

Two definite types, A and C, are described, and an intermediate or involution form B. Type A is the form of organism which is found in primary cultures derived from carcinomata. Type C occurred in association with A in one of the seven cases which were used in this research. The organism Type A is spherical, and varies in size from a minute dot to a diameter of $3\mu$. It grows on agar as a viscid, translucent streak. The organism Type C is spherical, with a diameter of 5 to $10\mu$, has a capsule, cell-network, and refractile granules, also nuclear bodies staining by Romanowski's method. It multiplies readily by budding when grown upon glucose agar. After inoculation it may multiply by sporulation. Type A never develops into Type C in vitro, but when injected into animals Type A (1) may not show morphological change, (2) may form clubs and ovals, or (3) may pass into the form of Type C. Only once were cultures of Type C obtained from animals inoculated with cultures of Type A. But either type can be preserved in full activity if inoculated from animal to animal in series. Cultures of Type C when inoculated into animals may remain unchanged or develop a process of sporulation resulting in Type A.

Type A is demonstrated in the tissues by Gram's method of staining, is found characteristically as an intracellular extranuclear parasite, and is usually single lying in a vacuolar "halo." These parasites are found in epithelial and endothelial cells and are not numerous.

When a culture of Type C is inoculated into the peritoneal cavity of an animal the organism can be demonstrated in the interstices between the proliferating cells (which follow the injection of either type). The parasite multiplies actively by budding and compresses the proliferating endothelium, and ultimately the nodules formed consist of masses of the organism with a network of connective-tissue-like appearance round and between them.

When cultures of Type A were injected into the peritoneal cavity of guinea-pigs and dogs the effects were uniform in those cases in which a positive result was obtained. The organism showed a capacity for initiating active proliferation of endothelial and epithelial cells. The new-formed tissue showed parenchyma and stroma, and at its edge a progressive growth without encapsulation. In some cases the neighbouring glands were the seat of new tissue deposits in structure resembling the primary nodules. Liver nodules were numerous in one of the cases and showed a structure exactly similar to the primary and glandular growths. This was apparently true visceral matastasis. As a rule when nodules appeared in the viscera of inoculated animals they seemed to arise from epithelium in loco. In the parenchyma cells of carcinomatous tumours of the female breast these parasites have been demonstrated. They occurred in each specimen examined, and were, as in the experimentally-formed nodules, not numerous. This organism was isolated from 58·3 per cent. of specimens of carcinoma mammæ examined. It has a definite life history consisting of two cycles, one vegetative budding cycle, the other a sporulating cycle.

The evidence derived from this research points to the conclusion that the organism described is an etiological factor in the morbid process known as carcinoma mammæ.

[1] Brit. Med. Jour. Jan. 23, 1904.

